# An overview of *Salmonella enterica* metal homeostasis pathways during infection

**DOI:** 10.1093/femsml/uqab001

**Published:** 2021-04-12

**Authors:** Olivier Cunrath, Jacob D Palmer

**Affiliations:** Department of Zoology, University of Oxford, Zoology Research and Administration Building, 11a Mansfield Rd, Oxford, UK OX1 3SZ; Department of Biochemistry, University of Oxford, New Biochemistry Building, 3 South Parks Road, Oxford, UK OX1 3PT; Department of Zoology, University of Oxford, Zoology Research and Administration Building, 11a Mansfield Rd, Oxford, UK OX1 3SZ; Department of Biochemistry, University of Oxford, New Biochemistry Building, 3 South Parks Road, Oxford, UK OX1 3PT

**Keywords:** metal homeostasis, nutritional immunity, Salmonella Typhimurium, infection, iron, magnesium

## Abstract

Nutritional immunity is a powerful strategy at the core of the battlefield between host survival and pathogen proliferation. A host can prevent pathogens from accessing biological metals such as Mg, Fe, Zn, Mn, Cu, Co or Ni, or actively intoxicate them with metal overload. While the importance of metal homeostasis for the enteric pathogen *Salmonella enterica* Typhimurium was demonstrated many decades ago, inconsistent results across various mouse models, diverse *Salmonella* genotypes, and differing infection routes challenge aspects of our understanding of this phenomenon. With expanding access to CRISPR-Cas9 for host genome manipulation, it is now pertinent to re-visit past results in the context of specific mouse models, identify gaps and incongruities in current knowledge landscape of *Salmonella* homeostasis, and recommend a straight path forward towards a more universal understanding of this historic host–microbe relationship.

## Teaser

A comprehensive overview of the importance of metal uptake and resistance mechanisms during *Salmonella* infections.

**ABBREVIATIONS**
ROS: Reactive Oxygen Species
TBDT: TonB-Dependent Transporter
NRAMP1: Natural Resistance Associated to Macrophage Protein 1 (also known as SLC11A1)
SLC11A1: Solute Carrier 11 A 1 (also known as NRAMP1)
DHBS: 2,3-dihydroxybenzoylserine
LCN2: Lipocalin 2

## INTRODUCTION

It has been nearly 80 years since Schade and Caroline reported that a component of egg white could bind with iron, preventing the growth of bacterial pathogens (Schade and Caroline 1944). Since that time, we have learned that indeed, during a bacterial infection, a host can employ a strategy known as nutritional immunity (Weinberg 1975; Hennigar and McClung 2016), where it can sequester essential nutrients, including biological metals such as Mg, Fe, Mn and/or Zn, by either secreting metal chelating agents like lipocalin or calprotectin (Sohnle *et al*. 1991; Flo *et al*. 2004; Corbin *et al*. 2008), or by actively removing the metals from the environment using metal pumps like NRAMP1 (Vidal *et al*. 1993; Wessling-Resnick 2015). However, preventing access to nutrients is not the only biological metal-based strategy, as the host can also actively *increase* metal concentrations of the environment, using proteins like ATP7A, thus causing Cu toxicity (White *et al*. 2009). In response to this host defence, pathogens have adapted highly sophisticated and well-regulated metal uptake and efflux mechanisms, allowing them to navigate the course of an infection while maintaining metal concentrations within the narrow, lethal boundaries of starvation and toxicity. While many of these bacterial metal homeostasis processes have been identified, their universal relevance can differ widely among pathogens.

*Salmonella* is a food-born pathogen that can cause both gastroenteritis and typhoid fever. More than a half-century ago, the discovery of a ‘Salmonellosis Resistance Factor’ found in fermented foods, demonstrated the enhanced ability for mice to survive a *Salmonella* infection (Schneider 1967). This resistance factor, originally coined ‘Salmonellosis pacifarin,’ and later determined to be the iron-chelating molecule, enterobactin/salmochelin (thoroughly discussed in the Iron section), established the first causal link of the necessity for a biological metal during a systemic infection (Wawszkiewicz *et al*. 1971). Through the many years since, considerable effort has been invested in understanding the biological metal uptake, efflux and regulatory elements in *Salmonella*. This ground-breaking body of work demonstrated the immense complexity of metal availabilities across mammalian tissues as well as its necessity for pathogenesis during infection (Diaz-Ochoa *et al*. 2016; Sassone-Corsi *et al*. 2016; Huang *et al*. 2017; Frawley *et al*. 2018; Cunrath and Bumann 2019). Despite these successes, however, experimentation to reveal the relative importance of each of the biological metals have utilized highly variable mouse models, genetically different *Salmonella* genotypes and various modes of infection, which have subsequently delivered occasional contradictory and confusing results.

It is therefore prudent to review and re-evaluate our current understanding of metal homeostasis during *Salmonella* infection in the context of the various *in vivo* experimental strategies.

### Magnesium (Mg)

Magnesium (which unlike the other metals discussed here, is not a d-block metal) is the most abundant divalent metal ion in bacteria (Outten and O'Halloran 2001; Cunrath *et al*. 2016). When complexed to water, Mg(II) has the unique quality of having a 400 times larger hydration sphere compared to its ionic sphere. Mg(II) mainly interacts with biological molecules, like DNA and proteins, through its hydration sphere rather than directly. One prominent exception is the interaction between ATP and Mg(II) where the oxygen of the phosphate group directly interacts with the Mg(II) ion. In hosts, Mg(II) blood levels are around 0.85 to 1.10 mM (Chernecky and Berger 2013), but free Mg(II) concentrations can vary depending on the cell compartment. While free concentrations are of around 0.5 to 1 mM in the cytosol, as well as in *Salmonella*-containing vacuoles early after phagocytosis (Martin-Orozco *et al*. 2006), phagosomal Mg(II) levels can drastically decrease and cause growth restricting deprivation. The divalent metal pump NRAMP1 (also known as SLC11A1) was shown to cause Mg(II) deprivation resulting in drastic proliferation defects for pathogens (Cunrath and Bumann 2019). The exact mechanism of inhibition remains to be determined, but NRAMP1 likely restricts Mg(II) access by either modulating fusion of *Salmonella* containing vacuoles with Mg(II)-containing vesicles, or by directly transporting Mg(II) outside the phagosome, similar to some bacterial NRAMP1 orthologs (Shin *et al*. 2014).

#### Mg uptake

Bacterial cells contain around 20–100 mM Mg(II) *in vitro* (Outten and O'Halloran 2001; Cunrath *et al*. 2016) and this relatively high abundance reflects its importance in bacterial physiology. Mg(II) plays a crucial role in lipid membrane stability, ribosome stability and activity; and is also a co-factor to several enzymes, requiring *Salmonella* to have high efficiency Mg(II) uptake systems. Due to its large hydration sphere, Mg(II) cannot be taken up by narrow cation channels used for similar metals such as Ca(II), Na(I) and K(I).

**Figure 1.**
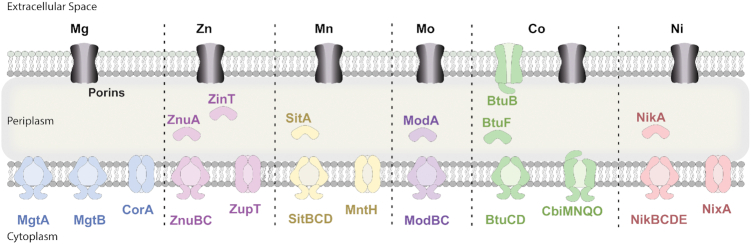
Proteins involved in divalent metal uptake (excluding Fe) in *Salmonella enterica* Typhimurium. For details see text. Porins are shown in grey. TBDT: BtuB. ABC-transporter: MgtA, MgtB, ZnuBC, SitABCD, ModBC, BtuCD and NikBCDE. Permease: CorA, ZupT, MntH and NixA. Periplasmic binding proteins: ZnuA, ZinT, SitA, ModA, BtuF and NikA.

Mg(II) is transported from the periplasm into the cytoplasm by two ABC transporters, MgtA and MgtB, with affinities of 29 μM and 6 μM (Snavely *et al*. 1989), respectively, or by the permease CorA with an affinity of 15 μM (Fig. 1). While *corA* is thought to be constitutively expressed, *mgtA* and *mgtB* expression is governed by periplasmic and cytoplasmic Mg(II) abundance. Indeed, low periplasmic Mg(II) concentration induces *mgtA* and *mgtB* expression through PhoPQ (which is also activated by acidic pH and antimicrobial peptides), while high cytoplasmic free Mg(II) concentration inhibits *mgtA* and *mgtB* expression transcriptionally via the proline-rich leader peptides, and post-translationally by direct binding to the transporter. Furthermore, in high Mg(II) conditions, the small regulatory peptide MgtR directly binds MgtA and induces MgtA proteolysis. (Hmiel *et al*. 1986; Snavely *et al*. 1989). MgtB is located on the highly regulated operon *mgtCBRcigR*, together with MgtC, a protein inhibiting the F_1_F_0_ATP synthase (reducing ATP levels and thus liberating cytoplasmic Mg(II)), MgtR, a small regulatory peptide, and CigR, an anti-virulence protein (for more on *mgtCBRcigR*, (Lee and Lee 2015; Park *et al*. 2019)).

The deletion of the main regulator, PhoPQ, has been shown to drastically decrease *Salmonella*’s ability to proliferate *in vivo* (Fields *et al*. 1989), henceforth referred to as a decrease in bacterial fitness or within-host fitness (Wiser and Lenski 2015). While PhoPQ is responsible for the expression of a vast number of virulence genes, the exact reason for this strong attenuated phenotype still remains to be elucidated, and the altered Mg(II) uptake could only be one of many effects. Contrarily, the deletion of the small regulatory peptide MgtR did not affect fitness (Lee and Groisman 2010). Although MgtA and MgtB are both dispensable in NRAMP1 negative mice singly, the double mutant is highly attenuated, which suggests that in NRAMP1 negative mice, one transporter can compensate the absence of the other (Table 1). In NRAMP1 positive mice, however, MgtB can rescue a MgtA mutant, while MgtA is unable to perform the reciprocal rescue for a MgtB mutant, indicating that MgtB is the main Mg(II) transporter *in vivo* (Moncrief and Maguire 1998; Cunrath and Bumann 2019). Expectedly, deletion of both transporters, MgtA and MgtB, results in a complete fitness defect, demonstrating that the permease CorA cannot sufficiently supply Mg(II) during systemic infection, though a *corA* deletion strain was shown to have attenuated fitness during an oral infection (Papp-Wallace *et al*. 2008).

**Table 1.** *In vivo* phenotype of Mg homeostasis mutants.

Gene	Description	Mutants	*Salmonella* genotype	Mouse model	Phenotype	Reference
*mgtA*	ABC transporter	*mgtA::MudJ*	ATCC14028	BALB/c; *i.p*.	None	(Blanc-Potard and Groisman 1997)
		*mgtA::MudCam mgtCB::MudJ*	ATCC14028	BALB/c; *i.p*	Attenuated	(Blanc-Potard and Groisman 1997)
		Δ*mgtA*	ATCC14028	C3H/HeN; *i.p*.	None	(Choi *et al*. 2019)
		*mgtA^P550,551A^*	ATCC14028	C3H/HeN; *i.p*.	Increased	(Choi *et al*. 2019)
		*mgtA^D377A^*	SL1344	various; *i.v*.	None	(Cunrath and Bumann 2019)
		*mgtA^D377A^mgtB^D379A^*	SL1344	various; *i.v*.	Attenuated	(Cunrath and Bumann 2019)
*mgtB*	ABC transporter	*mgtB::MudJ*	ATCC14028	BALB/c; *i.p*.	None	(Blanc-Potard and Groisman 1997)
		*mgtCB::MudJ mgtA::MudCam*	ATCC14028	BALB/c; *i.p*	Attenuated	(Blanc-Potard and Groisman 1997)
		Δ*mgtB*	ATCC14028	C3H/HeN; *i.p*.	Attenuated	(Choi *et al*. 2017)
		*mgtB^P555,556A^*	ATCC14028	C3H/HeN; *i.p*.	Increased	(Choi *et al*. 2017)
		Δ*mgtB*	SL1344	various; *i.v*.	Attenuated*	(Cunrath and Bumann 2019)
		*mgtB^D397A^*	SL1344	various; *i.v*.	Attenuated*	(Cunrath and Bumann 2019)
		*mgtB^D379A^mgtA^D377A^*	SL1344	various; *i.v*.	Attenuated	(Cunrath and Bumann 2019)
*corA*	permease	*corA::Tn10Δ16Δ17*	SL1344	BALB/c; oral	Attenuated	(Papp-Wallace *et al*. 2008)
*mgtR*	regulator	*mgtR::Cm*	ATCC14028	C3H/HeN; *i.p*.	None	(Lee and Groisman 2010)

* Attenuation was only observed in NRAMP1 positive mice, but not in NRAMP1 negative.

#### Mg resistance

**Figure 2.**
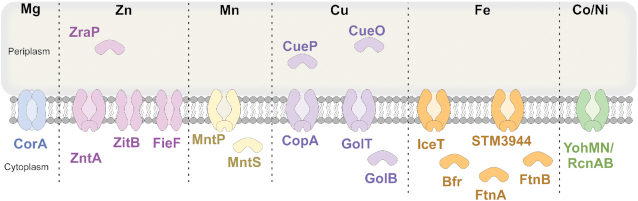
Proteins involved in divalent metal resistance mechanisms in *Salmonella enterica* Typhimurium. For details see text. Periplasmic binding proteins: ZraP, CueP and CueO. Cytoplasmic protein: MntS, GolB, Bfr, FtnA and FtnB. Permease: CorA. P-type ABC efflux transporter: ZntA, MntP, CopA, GolT, IceT, STM3944 and YohMN/RcnAB. Cation diffusion family protein: FieF and ZitB.

To avoid high accumulation of Mg(II) inside the bacterial cell, CorA can also efflux Mg(II) from the cytoplasm to the periplasm when Mg(II) concentrations are high (Snavely *et al*. 1989) (Fig. 2). CorB, CorC and CorD, also contribute to Mg(II)-efflux in a CorA-dependant manner (Gibson *et al*. 1991), but their exact role remains unknown. As mentioned, *corA* deletion has an attenuated phenotype during an oral infection, but it is impossible to confidently say whether it is due to its role in Mg(II) influx or -efflux.

#### Concluding remarks

Taken together, Mg(II) is an essential nutrient for *Salmonella* survival and appears to be *Salmonella*’s ‘Achilles’ heel’ when facing NRAMP1 induced magnesium limiting conditions *in vivo*. While the Mg(II) transporter MgtB is essential for full fitness, *Salmonella* appears to fine-tune its physiology to adapt to these starvation conditions. Additionally, Mg(II) toxicity seems to need further investigation to be conclusive.

### Iron (Fe)

Iron is a highly abundant transition metal that primarily exists in one of two redox states under physiological conditions, either as ferrous iron, Fe(II), or ferric iron, Fe(III). Despite its broad abundance in the earth's crust, most iron is found as a component of rather insoluble oxyhydroxide polymers, resulting in free Fe(III) in aerobic aqueous environments of approximately 10^−17 ^M, significantly lower than the essential intracellular iron requirement for many microbes (10^−6 ^M–10^−8 ^M) (Guerinot 1994). Total iron stores in the human body range from approximately 0.3 to 0.8 g in healthy adults and whole blood levels are around 9 mM (Cook *et al*. 1986; Blazewicz *et al*. 2013), yet free Fe(II) is kept under tight control, due to its hydroxyl radical generating potential via reactivity with H_2_O_2_ in the Fenton reaction (Fenton 1894; Halliwell and Gutteridge 1984; Touati 2000). During an infection, the host further decreases iron availability via increased ferroportin expression, which reduces serum iron levels, and lipocalin 2 (Lcn2) secretion, which can sequester some siderophores, such as enterobactin (Flo *et al*. 2004; Yeh *et al*. 2004). Intravacuolar iron availability is also decreased upon phagocytosis by the divalent metal pump NRAMP1 (Wessling-Resnick 2015).

#### Iron uptake

**Figure 3.**
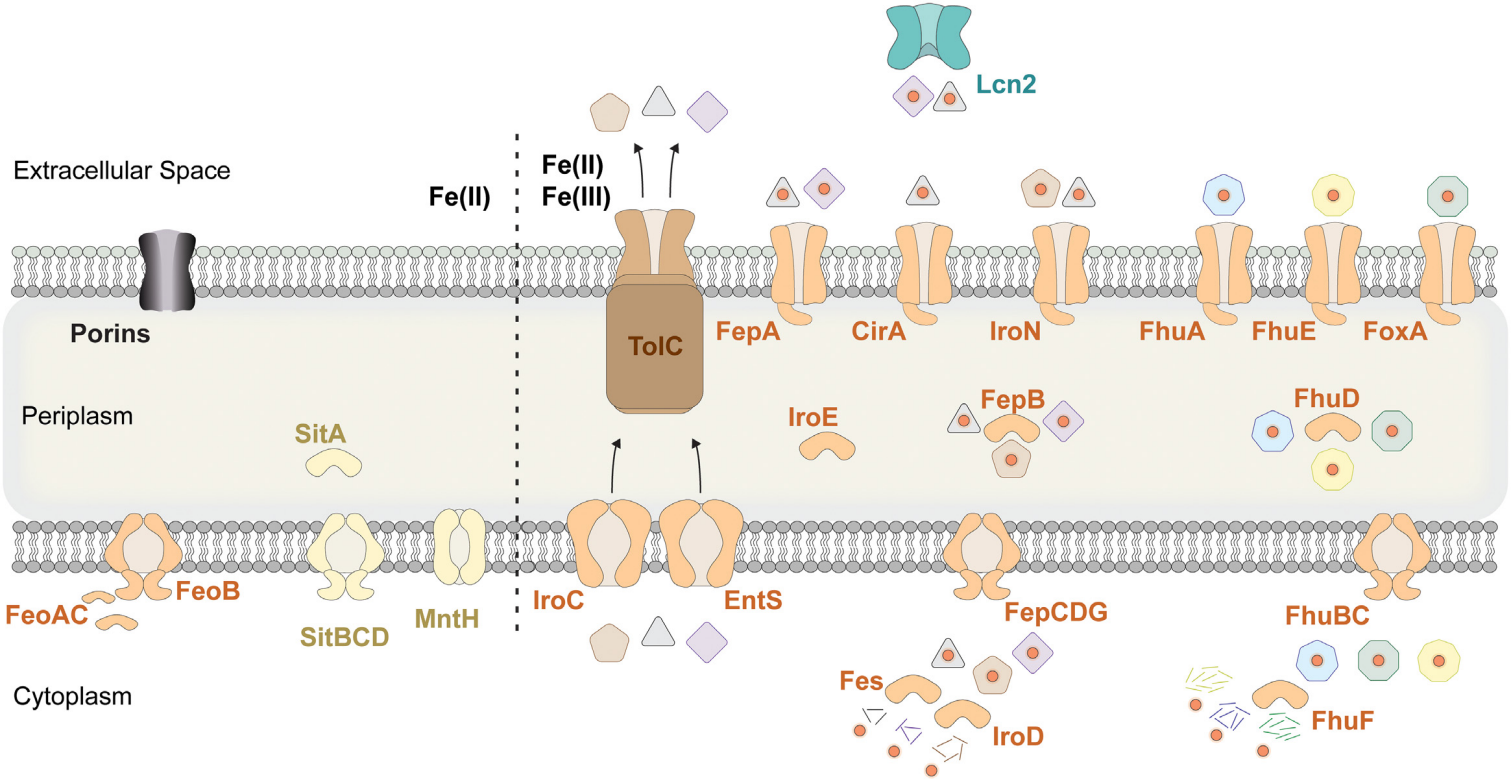
Proteins involved in Fe uptake in *Salmonella enterica* Typhimurium. For details see text. Siderophores DHBS (black triangle), enterobactin (purple square) and salmochelin (brown pentagram) are secreted by the ABC transporter IroC and EntS coupled to the outer membrane channel TolC. Lipocalin 2 (Lcn2 - cyan) sequesters DHBS and enterobactin in the extracellular space. Iron (orange circle) is captured and ferri-siderophores are transported into the cell via TBDT. Xeno-siderophores: ferrioxamine (dark green), ferrichrome (light blue) and ferricoprogen (yellow). TBDT: FepA, CirA, IroN, FhuA, FhuE and FoxA. ABC transporter: FeoB, SitBCD, FepCDG and FhuBC. Permease: MntH. Periplasmic binding protein: SitA, IroE, FepB and FhuD. Cytoplasmic protein: FeoAC, Fes, IroD, FhuF.

Iron plays a key role in the virulence of *Salmonella*, as is the case with many other pathogens (Doherty 2007; Nairz *et al*. 2010; Skaar 2010; Zughaier and Cornelis 2018), and *Salmonella* has been demonstrated to differentially regulate expression of up to 7% of its genome in response to environmental iron concentrations (Bjarnason *et al*. 2003). Iron can be imported via one of two primary routes in *Salmonella*, either bound by an iron-chelating siderophore as Fe(III)/Fe(II), or as a free Fe(II) cation (Fig. 3).

#### Ferric iron uptake

**Figure 4.**
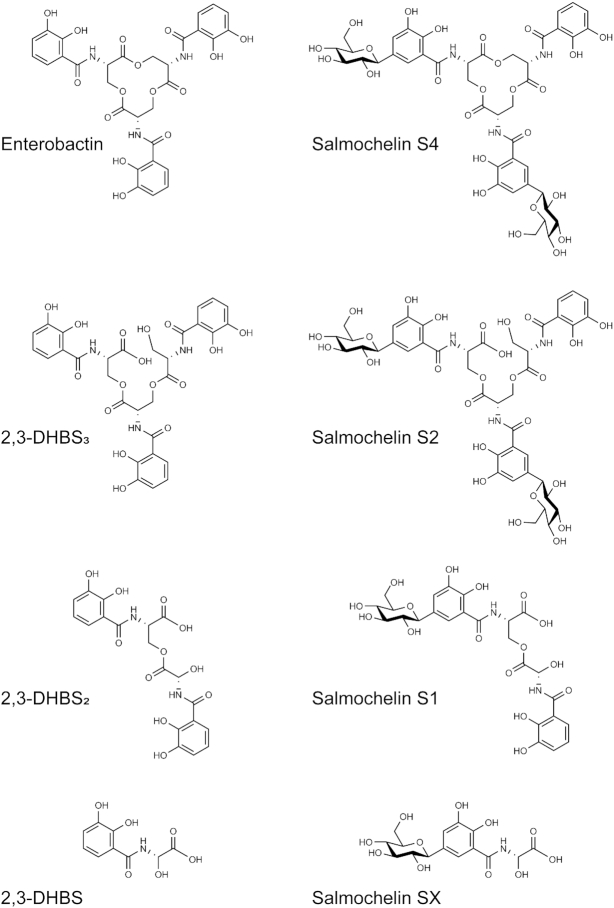
Chemical structures of siderophores produced by *Salmonella enterica* Typhimurium. For details see Fe section.

*Salmonella* produces its own siderophores, but can also utilize xenosiderophores (siderophores produced and secreted by other microbes). *Salmonella* uses its *ent* gene cluster (*entABCDEFHS*) responsible for enterobactin (also referred to as enterochelin) biosynthesis and export (Raymond *et al*. 2003). Enterobactin is the strongest known biological chelator of iron, (K_a_ = 10^52^ M^−1^ (Harris *et al*. 1979)) however, during immune trigged inflammation, enterobactin (and its breakdown product 2,3-dihydroxybenzoylserine; DHBS) can be sequestered in the host by Lcn2. Interestingly, *Salmonella* can evade this immune system driven iron-deprivation by converting enterobactin to salmochelin (Fischbach *et al*. 2006; Raffatellu *et al*. 2009) via C-glycosylation (IroB) (Fischbach *et al*. 2005) and linearization (IroE) (Zhu *et al*. 2005; Lin *et al*. 2005) (Fig. 4). After biosynthesis, EntS mediates enterobactin and DHBS secretion to the periplasm while IroC mediates enterobactin, DHBS and salmochelin secretion, which is then processed out of the periplasm by TolC (Hantke 2003; Crouch *et al*. 2008). Interestingly, *iroC entS* double mutants are still capable of secreting the siderophore breakdown products DHBS_2_, DHBS, and salmochelin SX, and recent work suggests it may be via the multi-drug efflux pump MacAB (Crouch *et al*. 2008; Bogomolnaya *et al*. 2020).

Entry of ferric siderophores is an energy dependent mechanism requiring a TonB-dependent transporter (TBDT), a periplasmic or cytoplasmic hydrolase and an inner membrane transporter. Ferric salmochelin, enterobactin and DHBS, require recognition by the TBDT FepA (enterobactin; DHBS), IroN (salmochelin; DHBS), and/or CirA (DHBS). After translocation to the periplasm, ferric siderophores are either bound by FepB, and then processed into the cytoplasm by the multi-subunit inner membrane permease, FepCDG (Wilkins and Lankford 1970; Lundrigan and Kadner 1986; Hantke 1990; Shea and McIntosh 1991; Baumler *et al*. 1998; Rabsch *et al*. 1999; Rabsch *et al*. 2003) or directly processed by the periplasmic hydrolase IroE. In the cytoplasm, ferric-siderophores are dissociated by the hydrolases Fes (enterobactin) or IroD (salmochelin). Differential substrate specificity for Fes and IroD explains the otherwise apparent functional redundancy (Lin *et al*. 2005). *Salmonella* can also utilize three xenosiderophores, ferrichrome (produced by fungi like *Aspergillus*) coprogen (produced by fungi like *Penicillium*), and ferrioxamine (produced by bacteria like *Streptomyces*). These xenosiderophores are taken up via the TBDT FhuA (ferrichrome) and FoxA (ferrioxamine) and both are further transported by the periplasmic binding protein FhuD, and the inner membrane complex FhuBC responsible for transporting them into the cytoplasm where the ferric iron is reduced by FhuF (Luckey *et al*. 1972; Fecker and Braun 1983; Sauer *et al*. 1987; Koster 1991; Kingsley *et al*. 1999; Matzanke *et al*. 2004).

Enterobactin, salmochelin and DHBS biosynthesis as well as siderophore transport and degradation, are all transcriptionally regulated by the Ferric uptake regulator (Fur), a master repressor protein that recognizes a canonical ‘Fur-box’ and represses expression of downstream genes when cytoplasmic iron levels are sufficient (Bagg and Neilands 1987; Baumler *et al*. 1996; Baumler *et al*. 1998; Lee and Helmann 2007; Troxell *et al*. 2011). Additionally, recent work has demonstrated post-transcriptional regulation of many enterobactin- and salmochelin-related genes by CsrA, a global stress response post-transcriptional regulatory protein (Romeo *et al*. 1993; Potts *et al*. 2017; Pourciau *et al*. 2019; Potts *et al*. 2019). IroN, has been shown to also be post-transcriptionally regulated by the small RNAs RhyB1 (also referred to as RfrA) and RhyB2 (also referred to as RfrB) (Masse and Gottesman 2002; Padalon-Brauch *et al*. 2008; Kim and Kwon 2013; Oglesby-Sherrouse and Murphy 2013; Balbontin *et al*. 2016).

The importance of siderophores during *Salmonella* infection has been widely studied, yet there remain open questions (Table 2). In the context of oral infection, *Salmonella* siderophore biosynthesis or uptake mutants have almost exclusively been reported as attenuated across multiple *in vivo* models. Common mutants of this variety include knockouts of *tonB* (Tsolis *et al*. 1996; Costa *et al*. 2016), *entC* (Crouch *et al*. 2008; Nagy *et al*. 2013), and the outer membrane receptors *iroN*, *fepA*, and *cirA* (Rabsch *et al*. 2003; Nagy *et al*. 2013). Additionally, to assess the importance of the individual siderophores, one early report generated an *iroBC* knockout, and therefore deficient in salmochelin biosynthesis, but also containing mutations in both *iroN* and *fepA*, leaving only *cir* for siderophore (primarily DHBS) uptake (Rabsch *et al*. 2003). This knockout mutant, along with other receptor protein knockouts, led the authors to conclude that both enterobactin and salmochelin are non-essential for full fitness during an oral infection of BALB/c mice, and that DHBS alone is sufficient (Rabsch *et al*. 2003). The importance of siderophores during gut colonization comes as no surprise, as many other enteric pathogens also produce siderophores for essential iron acquisition (Rutz *et al*. 1991; Thulasiraman *et al*. 1998), yet, for decades, the role of siderophores during systemic infection has been unclear, as different *in vivo* models have delivered varied results. In light of early reports that enterobactin was essential for full fitness in mice infected intraperitoneally (Yancey *et al*. 1979), a comprehensive study with multiple inbred and outbred mouse lineages, multiple enterobactin biosynthesis mutants, and using both intraperitoneal and intravenous route of infection, all showed full fitness for all mutants in all conditions (Benjamin *et al*. 1985). This remains the most comprehensive single study addressing variability in mouse lineage and mode of infection, yet the *Salmonella* mutants generated were selected based on growth defects, rather than an understanding of the genes disrupted, leaving some open questions regarding exactly which genes were interrupted that subsequently produced these results, and the 35 years since this study have not completely clarified the issue. Experiments with *E. coli* and Lcn2 deficient mice demonstrated that Lcn2 strongly sequesters enterobactin, and loss of Lcn2 resulted in massive increases in bacterial burden and bacteremia during intraperitoneal infection (Flo *et al*. 2004). Subsequent work with *iroA*^+^*E. coli* and *Salmonella* clearly demonstrated that salmochelin biosynthesis is crucial for full virulence, based on evasion of host-produced Lcn2, using both Lcn2^+^ and Lcn2^−^ C57BL/6 mice and both oral and intraperitoneal infection routes (Fischbach *et al*. 2005; Raffatellu *et al*. 2009; Nairz *et al*. 2015). Furthermore, siderophore biosynthesis and secretion mutants supplied intraperitoneally in C3H/HeN (NRAMP1+) mice were also attenuated, based on both mouse death rates and *Salmonella* infection load at systemic sites (Crouch *et al*. 2008). Conversely, however, studies in BALB/c, 129SvJ, and C57BL/6 backgrounds (both NRAMP1+ and NRAMP1-), and utilizing mutants of *tonB* or *entC* all demonstrated no attenuation (Tsolis *et al*. 1996; Boyer *et al*. 2002; Cunrath and Bumann 2019), exemplifying the current state of uncertainty surrounding Fe(III) uptake and systemic infection.

**Table 2.** *In vivo* phenotype of Fe homeostasis mutants.

Gene	Description	Mutants	*Salmonella* genotype	Mouse model	Phenotype	Reference
*ent*	Enterobactin biosynthesis	*ent ^−^*	SR-11	CFW; *i.p*.	Attenuated	(Yancey *et al*. 1979)
		*ent-1*	SL1344	various; *i.v./i.p*.	None	(Benjamin *et al*. 1985)
		*ent-7*	SL1344	various; *i.v./i.p*.	None	(Benjamin *et al*. 1985)
		*entB::*Cm	ATCC14028	c3H/HeN; *i.p*.	Attenuated	(Crouch *et al*. 2008)
		*entC::*Kan	ATCC14028	c3H/HeN; *i.p*.	Attenuated	(Crouch *et al*. 2008)
		*entS::*Cm	ATCC14028	c3H/HeN; *i.p*.	None	(Crouch *et al*. 2008)
		*entS*::FR; *iroC*::FRT	ATCC14028	c3H/HeN; *i.p*.	Attenuated	(Crouch *et al*. 2008)
		*entC*::FRT; *iroN*::FRT; *fepA*::FRT	SL1344	129SvEvTac; oral	Attenuated	(Nagy *et al*. 2013)
		Δ*entC;* Δ*feoABC*	SL1344	various; *i.v*.	None	(Cunrath and Bumann 2019)
		Δ*entC*; Δ*feoABC;* Δ*sitABCD;* Δ*mntH*	SL1344	various; *i.v*.	Attenuated	(Cunrath and Bumann 2019)
*tonB*		*tonB::*Kan	ATCC14028	BALB/c ByJ; oral/*i.p*.	Att./None	(Tsolis *et al*. 1996)
		*tonB::*Kan*; feoB::*Tet	ATCC14028	BALB/c ByJ; *i.p*.	None	(Tsolis *et al*. 1996)
		*tonB::*Kan	N/R	129/SvJ; *i.v*.	None	(Boyer *et al*. 2002)
		*tonB::*Kan	ATCC14028	C57BL/6 (DSS); oral	None	(Costa *et al*. 2016)
		*tonB::*Kan; *feoB*::Tet	ATCC14028	C57BL/6 (DSS); oral	Attenuated	(Costa *et al*. 2016)
		Δ*tonB*; Δ*feoABC*	SL1344	various; *i.v*.	None	(Cunrath and Bumann 2019)
*cirA; fepA; iroN*	TonB-dependent, outer membrane siderophore receptor	*iroN::*pGP704*; fepA::*Tn*10*dTc	ATCC14028	BALB/c; oral	None	(Rabsch *et al*. 2003)
		*iroN::*pGP704*; fepA::*Tn*10*dTc; *iroBC::*Kan	ATCC14028	BALB/c; oral	None	(Rabsch *et al*. 2003)
		*cir::*MudJ*; iroN::*pGP704*; fepA::*Tn*10*dTc	ATCC14028	BALB/c; oral	Attenuated	(Rabsch *et al*. 2003)
		*iroN*::FRT	SL1344	129SvEvTac; oral	Attenuated	(Nagy *et al*. 2013)
		*fepA*::FRT	SL1344	129SvEvTac; oral	Attenuated	(Nagy *et al*. 2013)
		*iroN*::FRT;*fepA*::FRT	SL1344	129SvEvTac; oral	Attenuated	(Nagy *et al*. 2013)
		*iroN*::FRT;*fepA*::FRT; *entC*::FRT	SL1344	129SvEvTac; oral	Attenuated	(Nagy *et al*. 2013)
		*iroN*::pGP704	ATCC14028	C57BL/6 (DSS); oral	None	(Costa *et al*. 2016)
*iroBCDE*	Salmochelin biosynthesis and degradation	*iroBC::*Kan*; iroN::*pGP704*; fepA::*Tn*10*dTc	ATCC14028	BALB/c; oral	None	(Rabsch *et al*. 2003)
		*iroC*::FRT; *entS*::FRT	ATCC14028	c3H/HeN; *i.p*.	Attenuated	(Crouch *et al*. 2008)
*fhuABCDEF; foxA*	Xenosiderophores	*foxA* ^−^ (frameshift)	ATCC14028	BALB/c; oral/*i.v*.	Attenuated	(Kingsley *et al*. 1999)
		*foxA*::Kan	SL1344	BALB/c; oral/*i.v*.	None	(Makki 2003)
*fepBCDG*	Periplasmic binding protein	*fepB*::FRT	SL1344	129SvEvTac; oral	Attenuated	(Nagy *et al*. 2013)
*feoABC*	Inner membrane transporter	*feoB::*Tet	ATCC14028	BALB/c ByJ; oral/*i.p*.	None	(Tsolis *et al*. 1996)
		*feoB::*Tet*; tonB::*Kan	ATCC14028	BALB/c ByJ; *i.p*.	None	(Tsolis *et al*. 1996)
		*feoB::*Tet	N/R	various; *i.v*.	Attenuated	(Boyer *et al*. 2002)
		*feoB::*Tet*; mntH::*Cm	N/R	various; *i.v*.	Attenuated	(Boyer *et al*. 2002)
		*feoB::*Tet; *sitAD::*Sm	N/R	various; *i.v*.	Attenuated	(Boyer *et al*. 2002)
		*feoB::*Tet; *mntH::*Cm; *sitAD::*Sm	N/R	various; *i.v*.	Attenuated	(Boyer *et al*. 2002)
		*feoB*::Tet	SL1344	Sv129S6; oral	Attenuated	(Nagy *et al*. 2014)
		*feoB::*Tet	ATCC14028	C57BL/6 (DSS); oral	None	(Costa *et al*. 2016)
		Δ*feoABC;* Δ*tonB*	SL1344	various; *i.v*.	None	(Cunrath and Bumann 2019)
		Δ*feoABC;* Δ*entC*	SL1344	various; *i.v*.	None	(Cunrath and Bumann 2019)
		Δ*feoABC;* Δ*entC;* Δ*sitABCD;* Δ*mntH*	SL1344	various; *i.v*.	Attenuated	(Cunrath and Bumann 2019)
*ftnAB*	Cytoplasmic storage	*ftnA*::Kan	ATCC14028	c3H/HeN; *i.p*.	None	(Velayudhan *et al*. 2007)
		*ftnB*::Kan	ATCC14028	c3H/HeN; *i.p*.	Attenuated	(Velayudhan *et al*. 2007)
		*ftnA*::Kan; *bfr*::Cm	ATCC14028	c3H/HeN; *i.p*.	None	(Velayudhan *et al*. 2007)
		*ftnA*::Cm; *ftnB*::Kan; *bfr*::Cm	ATCC14028	c3H/HeN; *i.p*.	Attenuated	(Velayudhan *et al*. 2007)
*bfr;bfd*	Cytoplasmic storage	*bfr*::Cm	ATCC14028	c3H/HeN; *i.p*.	None	(Velayudhan *et al*. 2007)
		*bfr*::Cm; *ftnA*::Kan	ATCC14028	c3H/HeN; *i.p*.	None	(Velayudhan *et al*. 2007)
		*bfr*::Cm; *ftnA*::Cm; *ftnB*::Kan	ATCC14028	c3H/HeN; *i.p*.	Attenuated	(Velayudhan *et al*. 2007)
*dps*	Cytoplasmic storage	*dps*::Cm	ATCC14028	c3H/HeN; *i.p*.	Attenuated	(Velayudhan *et al*. 2007)
*fur*	Transcription repressor	*fur*::Amp	ATCC14028	c3H/HeN; *i.p*.	Attenuated	(Velayudhan *et al*. 2007)

N/R: no reference.

Early work on the importance of xenosiderophores reported strong attenuation of a *foxA* mutant via both oral and intravenous infection routes (Kingsley *et al*. 1999), yet subsequent work with more precise genetic methods was unable to recapitulate this result in multiple virulence challenge studies, using both oral and intravenous routes (Makki 2003).

#### Ferrous iron uptake

Free Fe(II) can diffuse from the environment into the periplasm of *Salmonella* via porins, where it can then be transported into the cytoplasm by a number of systems. FeoABC is a ferrous iron uptake system with strong affinity for Fe(II) (∼0.5 µM) (Hantke 1987; Velayudhan *et al*. 2000; Lau *et al*. 2016) where FeoB is an F-type NTPase (Kammler *et al*. 1993; Marlovits *et al*. 2002; Gomez-Garzon and Payne 2020). FeoA is a cytoplasmic protein whose role in transport is not fully understood, though is thought to interact with FeoB, and loss of FeoA has been reported to result in a reduction of iron uptake (Kammler *et al*. 1993; Hantke 2003; Cartron *et al*. 2006). FeoC is thought to be a transcriptional repressor that co-regulates the *feoABC* operon, along with Fur and Fnr (Kammler *et al*. 1993; Cartron *et al*. 2006), as well as CsrA (Potts *et al*. 2019). However, additional alternative mechanistic roles for FeoC have been proposed where FeoC forms metal complexes with iron-sulfur clusters (Hung *et al*. 2012), or interacts directly with FeoB and protects it from proteolysis (Kim *et al*. 2013; Kim *et al*. 2015). MntH, SitABCD and ZupT can also serve as inner membrane importers of periplasmic Fe(II) (Makui *et al*. 2000; Kehres *et al*. 2002; Grass *et al*. 2005; Haemig *et al*. 2010), though with weaker affinities and thus are discussed more thoroughly in the Manganese (MntH and SitABCD) and Zinc (ZupT) sections.

The role of Fe(II) uptake has also been extensively studied in both oral and systemic routes of infection, though focus has primarily been on *feoB* and systemic route of infection (Table 2). As with Fe(III), various *in vivo* models have delivered conflicting results. Additionally, now that both Fe(II) and Fe(III) uptake has been discussed, mutant strains deficient in both systems will also be introduced, allowing for a more robust view regarding the role of iron. Early work utilizing BALB/c mice and intraperitoneal infection demonstrated full virulence for *Salmonella* mutants for both *feoB* and a double mutant *feoB tonB* (Tsolis *et al*. 1996), indicating that other iron acquisition systems can contribute to growth at systemic sites. Recently, Δ*feoABC* Δ*tonB* and Δ*feoABC* Δ*entC* mutants demonstrated similar results in C57BL/6 mice (both NRAMP1+ and NRAMP1-) infected intravenously (Cunrath and Bumann 2019). Conversely, however, studies in 129/SvJ (both NRAMP1+ and NRAMP1-) and Sv129S6 mice, using intravenous and oral infection, respectively, demonstrated attenuation in numerous *feoB* mutants (in isolation and in conjunction with *mntH*/*sitAD*) (Boyer *et al*. 2002; Nagy *et al*. 2014). A quadruple Δ*feoABC* Δ*entC* Δ*sitABCD* Δ*mntH* mutant, which lacks all known routes of iron uptake, was found to be strongly attenuated during systemic infection in both NRAMP1+ and NRAMP1- mice (Cunrath and Bumann 2019). While this result verifies the necessity of iron during a systemic infection, collectively there remain unresolved questions regarding the roles of ferrous and ferric iron during oral or systemic infection across various mouse models, and what *in vivo* models are most effective in determining fitness for these systems. Despite considerable focus on NRAMP1 and its clear capability to export Fe(II)/Fe(III), the iron uptake abilities of *Salmonella* are still capable of maintaining iron homeostasis within the macrophage during systemic infection (Cunrath and Bumann 2019).

#### Iron Resistance

While iron is essential for *Salmonella*, free Fe(II) can participate in the Fenton reaction and generate free hydroxyl radicals. As such, *Salmonella* has four distinct protein-based iron storage systems, called ferritins, which could be considered as iron resistance mechanisms, as they bind free cytoplasmic iron. Specifically, these four systems include FtnA and FtnB, which are canonical ferritins, Bfr, a bacterioferritin which contains heme, and Dps, a DNA-binding protein, which all sequester Fe(II) and store it as Fe(III) (Andrews *et al*. 1989; Almiron *et al*. 1992; Abdul-Tehrani *et al*. 1999; Andrews *et al*. 2003; Velayudhan *et al*. 2007). Ferritins and bacterioferritins are distantly related but are reported to be capable of storing more than 2000 iron atoms (Andrews 1998). Bfd is required to reduce Fe(III) stored in Bfr back to Fe(II) (Weeratunga *et al*. 2009; Yao *et al*. 2012). While mechanistic variability exists between these systems, they all serve the same functional purpose of sequestering free Fe(II) from the cytoplasm, safely storing it while environmental iron levels are sufficient, and then drawing upon these stores as environmental iron becomes limiting. *ftnA* and *bfr* were two of the first genes shown to be positively regulated by Fur (upregulated when iron levels are sufficient), based on post-transcriptional regulation by the antisense small RNA, RyhB2 (Masse and Gottesman 2002). In *E. coli*, CsrA was also shown to play a regulatory role in iron storage, but this has not yet been demonstrated in *Salmonella*.

The role of iron storage mechanisms during infection has also been investigated, albeit less extensively compared to the numerous studies focused on iron uptake (Table 2). *ftnB* and *dps* appear to play the biggest role during intraperitoneal infection of C3H/HeN mice, as both *Salmonella* mutants had attenuated phenotypes (Velayudhan *et al*. 2007). *ftnA* and *bfr* single and double mutants had no phenotype, yet a *ftnA*, *bfr*, *ftnB* triple mutant was more attenuated than the *ftnB* mutant alone, indicating that FtnA and Bfr may indeed play a role during infection (Velayudhan *et al*. 2007). This same study also investigated the role of *fur*, demonstrating the *fur* mutants were completely avirulent (Velayudhan *et al*. 2007).

In addition to these storage mechanisms, *Salmonella* also has the capability to export iron from the cytoplasm, at least under certain *in vitro* conditions (Fig. 2). First, IceT is a member of the MFS transporter superfamily, mediating efflux of citrate and iron-citrate, and regulated by the two-component system, BaeSR (Baranova and Nikaido 2002; Frawley *et al*. 2013). Additionally, overexpression of open reading frame *stm3944* led to reduced intracellular free iron, implicating it may play a role in iron efflux (Velayudhan *et al*. 2014). FieF (formerly YiiP) has also been shown to mediate iron efflux, though its expression is not regulated by Fur, and it is thought to be more directly involved with Zn(II) efflux (Grass *et al*. 2005; Wei and Fu 2006; Huang *et al*. 2018). The roles of IceT and STM3944 have not yet been investigated *in vivo*, though iron toxicity appears unlikely to occur *in vivo*.

#### Concluding remarks

Taken together, the importance of this essential nutrient is unquestionable. While the necessity of siderophores and more generally of iron uptake systems during gut colonization seem certain, there remains open speculation regarding the importance of the iron uptake systems during systemic infection. Recent findings showing that *Salmonella* high-affinity iron uptake systems are dispensable in maintaining iron homeostasis even in the presence of NRAMP1, challenged our current understanding. This new result, in conjunction with the long history of controversy, suggests that other mouse resistance loci (other than NRAMP1) and/or mechanisms that are of importance at later stage of the infection may be at the origin of conflicting results. Further investigation at the host level will be crucial for obtaining a unified understanding.

### Zinc (Zn)

In biological environments, zinc is a redox stable transition metal with a single oxidative state: Zn(II) (Irving and Williams 1953). Zn(II) has an incredibly high binding affinity with many proteins and is mainly complexed with histidine and cysteine (Andreini *et al*. 2008). Zinc blood levels in healthy adults is approximately 140 μM (Wastney *et al*. 1986; Blazewicz *et al*. 2013), but during an infection, the host can sequester free Zn(II) and other essential metal cations by secreting calprotectin, a divalent metal chelator, as a strategy to limit bacterial growth via nutrient starvation (Hood and Skaar 2012; Porcheron *et al*. 2013), as well as other systemic Zn(II) redistribution strategies (reviewed in (Haase and Rink 2014; Gammoh and Rink 2017)). A reciprocal strategy can also be employed by macrophages, where they utilize vesicular zinc to cause zinc toxicity of intracellular pathogens (Kapetanovic *et al*. 2016). However, macrophage-phagocytosed *Salmonella* has been shown to induce an increase in free Zn(II) levels in the cytoplasm of macrophages, as well as avoid colocalisation with zinc-containing vesicles, thus evading these key pathogen-clearing host defence mechanisms (Kapetanovic *et al*. 2016; Wu *et al*. 2017).

#### Zinc uptake

Approximately 6% of prokaryote proteins are proposed to be Zn(II)-binding (Andreini *et al*. 2008) and *Salmonella* is known specifically to utilize Zn(II) in many essential pathways like protein synthesis and DNA repair (Frawley *et al*. 2018). Intracellular zinc concentrations can vary between 0.2 and 2.5 mM, as determined by *E. coli* and *P. aeruginosa in vitro* measurements (Outten and O'Halloran 2001; Cunrath *et al*. 2016), suggesting that *Salmonella* requires efficient zinc uptake systems.

Zinc enters the periplasm most likely through outer membrane porins by passive diffusion, where it is sequestered by the Zn(II)-shuttling protein ZinT (Panina *et al*. 2003; Graham *et al*. 2009; Ilari *et al*. 2014). Subsequently, zinc is taken up by the ABC transporter ZnuABC, where ZnuA is a periplasmic binding protein, ZnuB is the integral membrane protein of the inner membrane, and ZnuC is the ATPase (Patzer and Hantke 1998) (Fig. 1). Various partial or complete *Salmonella znuABC* deletion mutants showed a significant decrease of fitness *in vivo* (Campoy *et al*. 2002; Ammendola *et al*. 2007; Petrarca *et al*. 2010; Liu *et al*. 2012; Cerasi *et al*. 2014; Cunrath and Bumann 2019), clearly demonstrating the necessity for Zn(II) during infection. ZinT forms a stable complex with ZnuA when bound to Zn(II), however, it was demonstrated to be dispensable during infection (Patzer and Hantke 1998; Petrarca *et al*. 2010; Ilari *et al*. 2014; Cunrath and Bumann 2019). In less Zn(II)-starved environments, *Salmonella* utilizes ZupT, which is an inner membrane permease of the ZIP (ZRT-, IRT-like protein) family that exhibits a broad substrate range and is constitutively expressed in *E. coli* (Grass *et al*. 2005). ZupT is capable of transporting many divalent cations (Fe(II), Co(II), Mn(II), and Cd(II)), yet competition experiments with other substrates demonstrated a preference for Zn(II) (Taudte and Grass 2010, Cerasi *et al*. 2014). ZupT is dispensable during gut colonisation (Cerasi *et al*. 2014), but the importance of ZupT during systemic infection remains controversial (Table 3). *zupT* mutants were shown to be systemically attenuated in DBA-2 mice (NRAMP^+^) but not in BALB/c mice (NRAMP^−^) (Cerasi *et al*. 2014). NRAMP alone is unlikely responsible for the observed differential attenuation, as the *mntH sitABCD zupT* triple mutant showed no decreased phenotype in genetically modified C57BL/6 NRAMP1^+^ or NRAMP1^+^ mice (Cunrath and Bumann 2019), suggesting consequential, yet unknown, genetic differences between DBA-2 and BALB/c mice (Karlinsey *et al*. 2010; Cerasi *et al*. 2014).

**Table 3.** *In vivo* phenotype of Zn homeostasis mutants.

Gene	Description	Mutants	*Salmonella* genotype	Mouse model	Phenotype	Reference
*zinT*	Periplasmic buffering	*zinT::Cm*	ATCC14028	BALB/c; *i.p*.	None	(Petrarca *et al*. 2010)
		*ΔzinT; znuA^Δ138^^–^^160^*	SL1344	various; *i.v*.	Attenuated^*1^	(Cunrath and Bumann 2019)
*znuABC*	ABC transporter	*znuC::Cm*	ATCC14028	BALB/c; oral/*i.p*.	Attenuated	(Campoy *et al*. 2002)
		*znuA::Kan*	ATCC14028	BALB/c; oral/*i.p*.	Attenuated	(Ammendola *et al*. 2007)
		*znuA::Kan*	ATCC14028	BALB/c; *i.p*.	Attenuated	(Petrarca *et al*. 2010)
		*znuABC::Kan*	ATCC14028	BALB/c; *i.p*.	Attenuated	(Petrarca *et al*. 2010)
		*znuA::Cm*	ATCC14028	C57BL/6; oral	Attenuated	(Cerasi *et al*. 2014)
		*znuA::Cm*	ATCC14028	various, oral	Attenuated	(Liu *et al*. 2012)
		*ΔzinT; znuA^Δ138^^–^^160^*	SL1344	various; *i.v*.	Attenuated	(Cunrath and Bumann 2019)
		*ΔznuABC*	SL1344	various; *i.v*.	Attenuated	(Cunrath and Bumann 2019)
*zupT*	ZIP-family transporter	*zupT::Kan*	ATCC14028	C3H/HeN; *i.p*.	Attenuated	(Karlinsey *et al*. 2010)
		*zupT::Kan*	ATCC14028	various; oral	None	(Cerasi *et al*. 2014)
		*zupT::Kan*	ATCC14028	various; *i.p*.	Attenuated	(Cerasi *et al*. 2014)
		*zupT::Kan*	ATCC14028	C3H/HeN; *i.p*.	Attenuated	(Karlinsey *et al*. 2010)
		*zupT::Kan; mntH::scar; sitA::tetRA*	ATCC14028	various; oral	Attenuated^*2^	(Diaz-Ochoa *et al*. 2016)
		Δ*zupT;* Δ*sitABCD;* Δ*mntH*	SL1344	various; *i.v*.	None	(Cunrath and Bumann 2019)
*zntA*	Efflux ATPase	*zntA::Apr*	4/74	C3H/HeN; *i.p*.	Attenuated	(Huang *et al*. 2017)
		*zntA::Cm zitB::Kan*	ATCC14028	C3H/HeOuJ; *i.p*.	Attenuated	(Frawley *et al*. 2018)
*zitB*	Cation diffusion exporter	*zitB::Apr*	4/74	C3H/HeN; *i.p*.	Attenuated	(Huang *et al*. 2017)
		*zntA::Cm zitB::Kan*	ATCC14028	C3H/HeOuJ; *i.p*.	Attenuated	(Frawley *et al*. 2018)
*zur*	Fur-family repressor	*zur::Cm*	ATCC14028	BALB/c; oral	None	(Campoy *et al*. 2002)
		*zur::Cm*	ATCC14028	BALB/c; *i.p*.	Attenuated	(Campoy *et al*. 2002)

*1 Attenuation may be due to *znuA^Δ138^^–^^160^*mutation.

*2 Attenuation was reported to be due to an impaired Mn uptake of this mutant in the ceacal content.

The zinc uptake regulator Zur, a Fur family repressor protein, binds DNA in the presence of intracellular Zn(II) with a very strong affinity, repressing expression of downstream genes, including *znuABC* and *zinT* (Patzer and Hantke 1998; Gilston *et al*. 2014). Loss of Zur, along with ZnuC (see above), demonstrated an attenuated phenotype in a mouse model of *Salmonella* infection (Campoy *et al*. 2002). Surprisingly, however, the single *zur* mutant demonstrated a higher LD_50_ compared to WT *Salmonella* only when challenged intraperitoneally, and not when challenged orally (Campoy *et al*. 2002), though further investigations are needed to confirm this result.

In addition to these high-affinity Zn(II) uptake systems, the ABC transporter, SitABCD, which functions primarily as an iron and manganese transporter in *Salmonella* (see manganese section), can effectively bind Zn(II) (Kehres *et al*. 2002), though strong evidence of transport has not been demonstrated. Finally, the controversial ZntB protein notably shares homology to the CorA family of Mg(II) transport proteins, and was first proposed to play a primary role in Zn(II) efflux (Worlock and Smith 2002; Caldwell and Smith 2003). However, more recent reports with full-length resolved structures of ZntB suggest that ZntB mediates Zn(II) uptake, rather than efflux (Gati *et al*. 2017). The role (if any) of ZntB in an infection model has not yet been investigated.

#### Zinc resistance

Zinc toxicity has been demonstrated to be caused by disruptive binding to iron-sulphur cluster proteins in *E. coli* (Li *et al*. 2019), and competition with Mn(II) binding sites in *Streptococcus* (McDevitt *et al*. 2011). While Zur is primarily implicated in regulation of Zn(II) uptake, ZraSR (formerly HydHG), and cytoplasmic regulator ZntR are the primary systems regulating Zn(II) resistance. ZraSR (STM4173–4174) is a two-component regulatory system (TCS), upregulating expression when excess periplasmic Zn(II) is detected by ZraS (Leonhartsberger *et al*. 2001) and upregulating the periplasmic stress response protein ZraP (Appia-Ayme *et al*. 2012). ZraP forms a stable homopolymer in the presence of Zn(II), serving as a periplasmic homeostasis chaperone by effectively chelating the cation (Appia-Ayme *et al*. 2012) (Fig. 2). The importance of the two periplasmic resistance systems ZraSR and ZraP during a *Salmonella* infection has not yet been elucidated.

In increasing Zn(II) concentrations, Zn(II) accumulates in the cytoplasm and is sensed by the cytoplasmic MerR-like regulatory protein ZntR, activating expression of the inner membrane transporter ZntA (Rensing *et al*. 1997; Yamamoto and Ishihama 2005; Wang *et al*. 2012). *Salmonella* has three separate inner membrane transporters involved in Zn(II) export: ZntA (P-type ATPase), and the cation diffusion family proteins ZitB and FieF (the latter also referred to as YiiP). An *E. coli* strain with disrupted *zntA* and *zitB* showed greater hypersensitivity to elevated Zn(II) levels as compared to wild-type (WT) and single mutants (Grass *et al*. 2001). FieF was shown to be a Zn(II)-transporting member of the cation diffusion family of exporters and is also capable of transporting Fe(II) and Cd(II) (Grass *et al*. 2005; Wei and Fu 2006). However, Zn(II) sensitivity assays revealed no difference between *fieF* mutant strains and WT, and overexpression of *fieF* provided no additional Zn(II) resistance (Grass *et al*. 2001). The ability to survive and cause systemic infection in C3H/HeN mice was significantly attenuated for mutants of *zntA* and *zntA zitB* but not *zitB* alone (Huang *et al*. 2017; Frawley *et al*. 2018), suggesting that Zn(II) efflux likely plays a role during systemic infection (Table 3).

#### Concluding remarks

Taken together, zinc is an essential micro-nutrient and *Salmonella* needs its strong affinity uptake transporter ZnuABC during enteric and systemic proliferation. While ZinT is dispensable, it remains controversial whether ZupT contributes to *in vivo* fitness. In light of the difference in zinc affinity (0.7 μM for ZupT compared to <20 nM for ZinT-ZnuABC) it may appear that ZnuABC is the most relevant transporter in Zn(II) starved environments. Additionally, zinc toxicity may also play a substantial role during systemic proliferation, suggesting various micro-environments with varying zinc availabilities.

### Manganese (Mn)

It is possible for manganese to exist in three different oxidation states in biological environments: Mn(II), Mn(III) and Mn(IV), though Mn(II) is the predominant state (Zhu and Richards 2017). Manganese blood levels are around 0.3 µM (Blazewicz *et al*. 2013), but the secretion of calprotectin by neutrophils heavily decreases divalent cation availability, including Mn(II). Additionally, it has been shown that NRAMP1 decreases the Mn(II) levels of the *Salmonella*-containing vacuole during systemic infection, making it a potentially low bio-available micronutrient (Wessling-Resnick 2015).

#### Mn Uptake

Manganese plays a role in many biological systems, though is often implicated in the oxidative stress response, as a cofactor with Mn-superoxide dismutase (SodA) to quench ROS (Tsolis *et al*. 1995) or in metabolic enzymes like the L-arabinose isomerase AraA (Manjasetty and Chance 2006).

While Mn(II) import through the outer membrane is mainly due to passive diffusion via porins, *Salmonella* possess two main Mn(II) inner membrane transporters, MntH and SitABCD (Fig. 1). MntH is a proton-dependent NRAMP1 homolog with an affinity of 0.1 μM for Mn(II) (Kehres *et al*. 2000; Kehres *et al*. 2002) and was shown to be upregulated in response to low Mn(II) and the presence of H_2_O_2_ (Kehres *et al*. 2000; Kehres *et al*. 2002). SitABCD is an ABC transporter and also mediates influx of Mn(II) with an affinity equal to that of MntH, where SitA is the periplasmic binding protein, SitB is an ATP-binding protein, and SitC and SitD are integral membrane permeases (Zhou *et al*. 1999). Interestingly, while MntH and SitABCD are both upregulated in response to low Mn(II) and have similar apparent affinity for Mn(II) regardless of pH, changes in pH drastically and inversely affect the transport rates of the two systems. MntH transports Mn(II) most effectively in acidic conditions while SitABCD is essentially non-functional, while at slightly alkaline pH, SitABCD is optimally functional and MntH rates decrease with increasing pH (Kehres *et al*. 2002). Both transporters have binding capacity for other biological transition metals, though Mn(II) seems to be the highly preferred substrate in biologically relevant conditions (Kehres *et al*. 2002).

There are currently significant gaps in understanding the roles of MntH and SitABCD during a *Salmonella* infection (Table 4). While *mntH* mutants are mainly dispensable or showing only a very slight attenuation *in vivo* (Kehres *et al*. 2000; Janakiraman and Slauch 2000; Boyer *et al*. 2002; Zaharik *et al*. 2004; Karlinsey *et al*. 2010; Nagy *et al*. 2014), *sitABCD* deletion mutants demonstrate an attenuated phenotype linked to the presence of NRAMP1 (Janakiraman and Slauch 2000; Boyer *et al*. 2002; Zaharik *et al*. 2004). However, recent work using competitive index with WT during intravenous coinfection showed no decreased fitness for a *mntH sitABCD zupT* mutant in both NRAMP1^+^ and NRAMP1^−^ mice, contradicting previous reports (Nagy *et al*. 2014; Cunrath and Bumann 2019).

**Table 4.** *In vivo* phenotype of Mn homeostasis mutants.

Gene	Description	Mutants	*Salmonella* genotype	Mouse model	Phenotype	Reference
*mntH*	Proton-dependent importer	*mntH::Kan*	LT2	BALB/c; oral	Attenuated	(Kehres *et al*. 2000)
		*mntH::Cm*	N/R	various; *i.v*.	None	(Boyer *et al*. 2002)
		*mntH::Kan*	SL1344	various; *i.p*.	Attenuated	(Zaharik *et al*. 2004)
		*mntH::Kan*	ATCC14028	C3H/HeN; *i.p*.	Attenuated	(Karlinsey *et al*. 2010)
		*mntH::Kan*	SL1344	129SvEvTac; oral	None*	(Nagy *et al*. 2014)
		Δ*mntH;* Δ*sitABCD;* Δ*zupT*	SL1344	various; *i.v*.	None	(Cunrath and Bumann 2019)
*sitABCD*	ABC transporter	*sitA::*Cm	ATCC14028	various; oral/*i.p*.	Attenuated	(Janakiraman and Slauch 2000)
		*sitABCD::Sm*	N/R	various; *i.v*.	Attenuated	(Boyer *et al*. 2002)
		*sitA::Cm*	SL1344	various; *i.p*.	Attenuated	(Zaharik *et al*. 2004)
		*sitA::Kan*	SL1344	129SvEvTac; oral	None*	(Nagy *et al*. 2014)
		Δ*sitABCD;* Δ*mntH;* Δ*zupT*	SL1344	various; *i.v*.	None	(Cunrath and Bumann 2019)
*mntP*	Efflux pump	Δ*mntP*	SL1344	various; *i.v*.	None	(Cunrath and Bumann 2019)

* No fitness defect was reported when infected singly, but strains were attenuated during co-infection.

N/R: no reference

In response to low Mn(II) levels, *mntH* and *sitABCD* are both upregulated by the negative regulator MntR, a DtxR-family Mn(II)-binding transcription factor. Additionally, it has been shown that the expression of *mntH* and *sitABCD* are also affected by the peroxide sensing regulator, OxyR, and by Fur (see Iron section) (Patzer and Hantke 2001; Kehres *et al*. 2002; Ikeda *et al*. 2005; Martin *et al*. 2015). MntR also regulates expression of a second Mn(II) regulatory protein: MntS. MntS is a small protein (42 amino acids) whose mechanistic role is incompletely understood, but knockout and mutation studies showed that MntS does not appear to interact directly with Mn(II), but instead inhibits the activity of the Mn(II) efflux pump, MntP (see below) (Martin *et al*. 2015). No studies have directly tested the role of MntR or MntS *in vivo*.

#### Mn resistance

Mn(II) toxicity is believed to be due to cation competition of Mg(II)- and Fe(II)-binding sites, leading to impaired energy metabolism (Hohle and O'Brian 2014). MntP is the Mn(II) P-type ABC efflux transporter in *Salmonella*, regulated transcriptionally by MntR and translationally by a Mn(II)-dependent riboswitch (Waters *et al*. 2011; Dambach *et al*. 2015) (Fig. 2). In the absence of MntP, manganese toxicity can occur, which has been proposed as being due to Mn(II) displacement of Fe(II) from its binding sites, resulting in an increase in free cellular Fe(II) (Guedon *et al*. 2003). A *mntP* knockout mutant demonstrated no decrease in fitness compared to WT during intravenous co-infection (Cunrath and Bumann 2019), suggesting that Mn(II) toxicity is not a stress *Salmonella* encounters during systemic proliferation (Table 4).

#### Concluding remarks

While manganese uptake seems to contribute to the *in vivo* fitness during gut colonisation, the importance of those systems during systemic infection is questionable. The recent findings showing that the main Mn(II) transporters are dispensable during systemic infection, regardless of the presence or absence of NRAMP1, challenge our current knowledge of the importance of manganese during pathogenesis. Additional loci, other than NRAMP1, present in C3H/HeN or 129/Sv mice or additional physiological changes at later time points of the infection, may further decrease Mn(II) availability by yet unknown mechanisms. However, at an early stage of the infection, Mn(II) uptake systems seem dispensable. This might be due to sufficient extracellular Mn (II) concentrations for unspecific uptake or Mn(II) might be of secondary importance for *Salmonella* physiology during early systemic proliferation. The latter hypothesis is compatible with the fact that no Mn(II)-dependent protein has yet been identified as essential for full *in vivo* fitness.

### Copper (Cu)

Copper is the third most abundant transition metal in bacteria and exists in biological environments in two oxidative states, Cu(I) and Cu(II) (Liochev and Fridovich 2002), each with different affinities for biological molecules. Cu(I) prefers to bind to sulphur donors such as cysteine and methionine while Cu(II) binds preferably to nitrogen or oxygen donors such as histidine, glutamate or aspartate (Irving and Williams 1953). Total copper concentration in healthy adults is around 17 μM (Blicharska *et al*. 2008). During an infection, free plasma copper concentration has been estimated to be around 0.1 pM (Linder and Hazegh-Azam 1996) but macrophages where shown to accumulate copper in their phagosome to intoxicate intracellular pathogens (White *et al*. 2009; Achard *et al*. 2012).

#### Copper uptake

All known proteins utilizing copper as co-factors are localized either in the periplasm or the inner membrane, such as the inner membrane cytochrome c oxidase subunit I CyoB (Chepuri *et al*. 1990) and the periplasmic CuZn-superoxide dismutase SodC (Farrant *et al*. 1997; UniProt 2019), suggesting that no cytoplasmic uptake is needed for *Salmonella*. *In vitro* measurements of *E. coli* and *P. aeruginosa* cells show that cellular copper concentration can vary between 20–300 μM (Outten and O'Halloran 2001; Cunrath *et al*. 2016).

Copper enters the periplasmic space through passive diffusion via porins. To date, no active copper-specific inner membrane uptake system has been identified. The absence of evidence of cytoplasmic copper proteins and the wide distribution of copper exporters in *Salmonella* allows us to suggest that *Salmonella* is trying to avoid any cytoplasmic copper.

#### Copper resistance

A major research focus for copper in biological systems relates to copper toxicity, which is due to three major mechanisms. Copper can (i) react with hydrogen peroxide, forming ROS through Fenton's reaction (Liochev and Fridovich 2002); (ii) bind non-specifically to proteins and other molecules, thus inhibiting their function (Irving and Williams 1953); and (iii) Cu(I) can replace Fe(II) in the Fe-S cluster, inhibiting the activity of Fe-S proteins and liberating iron that can cause further damage (Macomber and Imlay 2009).

To survive in high copper concentrations, *Salmonella* has several resistance mechanisms, including active efflux, oxidase and periplasmic binding proteins (Fig. 2), which provide it with a minimum inhibitory concentration (MIC) for CuSO_4_ of 8 mM in aerobic and 1 mM in anaerobic conditions (Arai *et al*. 2019). To sense periplasmic copper concentrations, *Salmonella* uses the two-component system (TCS) CopS/R, which has been shown to be dispensable during both enteritis and systemic infection (Yoon *et al*. 2009). However, the target genes of this TCS have not yet been identified in *Salmonella*, though it may positively regulate copper resistance proteins like CopA, as has been observed in *P. aeruginosa* (Quintana *et al*. 2017). Excess periplasmic Cu(II) is bound by the cupric binding protein, CueP, which helps deliver copper to copper-binding proteins like the periplasmic superoxide dismutase, SodCII (Osman *et al*. 2013). A *cueP* mutant was shown to have no significant fitness loss compared to WT *Salmonella* during systemic infection (Fenlon and Slauch 2017). Additionally, CueO (formely known as CuiD), a periplasmic multi-copper oxidase, oxidizes the more reactive Cu(I) into the less reactive Cu(II) (Lim *et al*. 2002). In addition to its cuprous oxidase activity (K*_m_* of 34.4 ± 12.7 μM), CueO has also been shown to have ferroxidase activity (K*_m_* 52.6 ± 18.1 μM) (Achard *et al*. 2010). A *cueO* mutant was first described as attenuated in systemic sites (Achard *et al*. 2010), while two other studies showed the mutation of *cueO* led to no significant decrease of fitness (Craig *et al*. 2013) and the quadruple mutant *cueO cueP copA golT*, lacking the four main copper resistance genes, had no significant fitness loss during systemic infection (Cunrath and Bumann 2019).

At high concentrations, copper might still enter the cytoplasm through a less specific general divalent cation transporter, like ZupT (Taudte and Grass 2010). Cytoplasmic copper is sensed by the high-sensitivity MerR-type copper-resistance regulators CueR and GolS, which induce target gene expression by responding to monovalent cations like Cu(I) and Au(I) (Changela *et al*. 2003; Checa *et al*. 2007; Ibanez *et al*. 2013). *E. coli* CueR affinity for copper has been measured at a K_d_ of 10^−21^ M^1^ (Changela *et al*. 2003) and *Salmonella* GolS and CueR seem to have similarly strong affinities for Cu(I) (Osman *et al*. 2013). Adaptation to oxidizing or acidic conditions and monovalent cation selectivity (GolS is more sensitive to Au(I) than CueR) are suggested to explain the apparent redundancy of GolS and CueR (Changela *et al*. 2003; Osman *et al*. 2013). In the cytoplasm, excess copper is sequestered by the chaperon protein GolB, which also binds Au(I) and is under the regulation of GolS, (Checa *et al*. 2007; Wei *et al*. 2015) and the two P-type ATPases (CopA and GolT) actively transport copper ions from the cytoplasm to the periplasmic space (Checa *et al*. 2007; Espariz *et al*. 2007), both under the regulation of CueR and GolS, respectively. Surprisingly, the deletion of the P-type Zn(II) efflux pump ZntA (see Zn section) was shown to decrease Cu resistance in *Salmonella* 4/74, suggesting a potential role in Cu efflux (Huang *et al*. 2017). The deletion of the locus STM0324–STM0360, containing *golTSB* (STM0348–0350), was shown to be have no significant fitness defect compared to WT *Salmonella* during systemic infection (Haneda *et al*. 2009). Furthermore, both CopA and GolT were shown to be dispensable during systemic infection (Fenlon and Slauch 2017; Cunrath and Bumann 2019), as mentioned above (Table 5).

**Table 5.** *In vivo* phenotype of Cu homeostasis mutants.

Gene	Description	Mutants	*Salmonella* genotype	Mouse model	Phenotype	Reference
*cueP*	Cupric binding protein	*cueP::Cm*	ATCC14028	various; *i.p*.	None	(Fenlon and Slauch 2017)
		*cueP::Cm* Δ*copA* Δ*golT*	ATCC14028	various; *i.p*.	None	(Fenlon and Slauch 2017)
		Δ*cueP* Δ*copA* Δ*golT* Δ*cueO*	SL1344	various; *i.v*.	None	(Cunrath and Bumann 2019)
*cueO*	Multi-copper oxidase	*cueO::kan*	SL1344	C57BL/6; oral	Attenuated	(Achard *et al*. 2010)
		*cueO::Cm*	ATCC14028	BALB/c; *i.p*.	None	(Fenlon and Slauch 2017)
		Δ*cueO* Δ*copA* Δ*golT* Δ*cueP*	SL1344	various; *i.v*.	None	(Cunrath and Bumann 2019)
*golB*	Cytoplasmic chaperon	*STM0324–STM0360::kan*	ATCC14028	BALB/c; *i.p*.	None	(Haneda *et al*. 2009)
*copA*	Efflux ATPase	Δ*copA* Δ*golT*	ATCC14028	various; *i.p*	None	(Fenlon and Slauch 2017)
		Δ*copA* Δ*golT* Δ*cueP* Δ*cueO*	SL1344	various; *i.v*.	None	(Cunrath and Bumann 2019)
*golT*	Efflux ATPase	Δ*golT* Δ*copA*	ATCC14028	various; *i.p*	None	(Fenlon and Slauch 2017)
		*STM0324–STM0360::kan*	ATCC14028	BALB/c; *i.p*.	None	(Haneda *et al*. 2009)
		Δ*golT* Δ*copA* Δ*cueP* Δ*cueO*	SL1344	various; *i.v*.	None	(Cunrath and Bumann 2019)
*golS*	Cytoplasmic regulator	*STM0324–STM0360::kan*	ATCC14028	BALB/c; *i.p*.	None	(Haneda *et al*. 2009)
*copSR*	Two-component system	*copSR::Cm*	ATCC14028	BALB/c; oral, *i.p*.	None	(Yoon *et al*. 2009)

#### Concluding remarks

Taken together, these results suggest that it is unlikely that *Salmonella* encounters toxic copper concentrations during systemic proliferation, but further investigations need to address the importance of these systems during enteritis.

### Molybdenum (Mo)

Molybdenum mainly pre-exists in biological environments in its highest oxidative state, Mo(VI), as molybdate (MoO_4_^2−^) (Williams and Frausto da Silva 2002). Molybdenum, along with tungsten (W), is used in bacteria in the organic cofactor, molybdopterin (occasionally called pyranopterin) (Hille *et al*. 2014). Unfortunately, there is scant data regarding molybdenum concentration in human tissues during infection, though it has been shown that total molybdenum levels of approximately 17 nM do not change during a tuberculosis infection (Oh *et al*. 2019).

#### Molybdenum uptake

Molybdenum can shift oxidative state from 4+ to 6+, and is commonly used as a key cofactor in metabolic transformation of sulfur, carbon and nitrogen compounds (Hille *et al*. 2014; Leimkuhler and Iobbi-Nivol 2016). *In vitro* measurements from *E. coli* and *P. aeruginosa* show that molybdenum concentration is around 10–30 μM (Schneider 1967; White *et al*. 2009). Molybdate is thought to enter the periplasm by diffusion through porins, where it is then transported through the inner membrane by the ABC transporter ModABC, in which ModA is the periplasmic binding protein, ModB the permease and ModC the ATPase (Walkenhorst *et al*. 1995) (Fig. 1). ModABC expression is under the negative control of ModE (formely ModR) which binds molybdate in low micromolar affinity range (K_d_ of 8.10^−7 ^M) (Grunden *et al*. 1996; Anderson *et al*. 1997). Additionally, it has been shown that ModA can bind tungsten (W), which has the potential to replace molybdenum in some molybdenum-containing enzymes (Hille *et al*. 2014; Zhu *et al*. 2018). No investigations regarding the importance of the Mod system during infection have been conducted.

#### Molybdenum resistance

Molybdate toxicity is mainly due to its strong potential to disrupt accurate DNA repair, and is thus often used for random mutagenesis (Flessel 1977). *Salmonella* can survive toxic concentrations of up to 1mM of Na_2_MoO_4_, though this concentration is highly unlikely to occur under physiological conditions (Oliver *et al*. 2010). To date, no molybdenum specific resistance mechanism has been identified.

### Cobalt (Co)

Cobalt pre-exists in the oxidative states Co(II) and Co(III), but in biological environments, it is mainly found as the co-factor, cobal(III)amin (vitamin B12), in which it can transit from Co(I) up to Co(III) (Barras and Fontecave 2011). Nothing is known about cobalt concentrations in human tissues during infection, but whole blood cobalt concentrations are around 0.6 μM in healthy individuals (Blazewicz *et al*. 2013).

#### Co uptake

Cobalt is used in vitamin B12, but may also be used as co-factor alone like in the Co(II) or Zn(II)-utilizing enzyme AroB (Bender *et al*. 1989). Vitamin B12 uptake has mainly been studied in *E. coli* and is thought to be similar in *Salmonella*. The *btu* genes encode BtuB, an outer membrane TBDT, BtuF, which is a periplasmic binding protein, and BtuCD, an inner membrane ABC transporter (Yang *et al*. 2018) (Fig. 1). Additionally, *Salmonella* possesses a specific cobalt ion inner membrane uptake system, *cbiMNQO*, which has been shown to specifically transport cobalt (Rodionov *et al*. 2006; Finkenwirth *et al*. 2020). The final mode of uptake falls to nonspecific divalent metal permeases, which have been shown to transport cobalt with relatively lower affinities, such as CorA (see Mg section) (Guskov and Eshaghi 2012), ZupT (see Zn section) (Grass *et al*. 2005) and MntH (see Mn section) (Kehres *et al*. 2002). Both the cobalt (*cbiMNQO*) and vitamin B12 (*btuB*) specific uptake systems have been shown to be dispensable during systemic infection (Sampson and Gotschlich 1992; Bjorkman *et al*. 1996; Cunrath and Bumann 2019) (Table 6).

**Table 6.** *In vivo* phenotype of Co homeostasis mutants.

Gene	Description	Mutants	*Salmonella* genotype	Mouse model	Phenotype	Reference
*cibMNQO*	ABC transporter	Δ*cbiMNQO*; Δ*tonB*	SL1344	various; *i.v*.	None	(Cunrath and Bumann 2019)
*btuB*	Vitamin B12 outer membrane transporter	*btuB::Tn10*	TT16729/LT2	BALB/c; *i.p*.	None	(Sampson and Gotschlich 1992, Bjorkman *et al*. 1996)
		Δ*tonB;* Δ*cbiMNQO*	SL1344	various; *i.v*.	None	(Cunrath and Bumann 2019)

#### Co resistance

Due to the well-known thiophilicity of cobalt ions, cobalt toxicity is mainly due to its ability to disrupt Fe-S cluster stability by replacing the Fe(II) ion (Barras and Fontecave 2011). Cobalt resistance in *Salmonella* is relatively understudied, but the *Salmonella* genome carries the cobalt/nickel resistance *yohLMN* cluster, previously identified in *E. coli* (also known as *rcnRAB*). Encoded within this cluster, YohMN/RcnAB is an efflux transporter providing nickel and cobalt resistance (Rodrigue *et al*. 2005) and YohL/RcnR is the cytoplasmic regulator (Li *et al*. 2020). It is believed that ZntA (See Zn section) may also play a role in the efflux cobalt (Chaoprasid *et al*. 2015) (Fig. 2). The contribution to *in vivo* fitness of the cobalt resistance genes has not yet been investigated.

### Nickel (Ni)

In biological environments, nickel commonly pre-exists as Ni(II). Total nickel concentration in whole blood of healthy adults is around 85 nM (Christensen *et al*. 1979), but nothing is known of nickel availability during infection. Proteins involved with nickel homeostasis and their contribution both *in vitro* and during infection have not yet been investigated in *Salmonella*.

#### Ni uptake

In *E. coli* and *P. aeruginosa*, the bacterial nickel concentration is around 20–40 μM (Schneider 1967; White *et al*. 2009) and the *Salmonella* genome harbours only a few identified nickel-containing enzymes, such as the glyoxalase, GloA (Reiger *et al*. 2015). The *Salmonella* genome carries (i) a putative NiCoT-family permase NixA (STM2783) which was described in *Helicobacter pylori* to be involved in Ni uptake (Fischer *et al*. 2016); (ii) a putative ABC transporter operon NikABCDE (STM1255) previously described in *E. coli* (Rodionov *et al*. 2006) and (iii) a cytoplasmic nickel-responsive regulator NikR (STM3584), also identified in *H. pylori* and *E. coli* (Rodionov *et al*. 2006; Vannini *et al*. 2017), suggesting the presence of nickel uptake system, though no experimental evidence has been reported (Fig. 1).

#### Ni resistance

Nickel toxicity has not been investigated in *Salmonella*, but *Salmonella* carries the nickel/cobalt resistance *yohLMN* cluster, previously identified in *E. coli*, in which YohM is an efflux transporter providing nickel and cobalt resistance (Rodrigue *et al*. 2005) (Fig. 2).

## CONCLUDING REMARKS AND PERSPECTIVES

In this review we highlight that *Salmonella* metal homeostasis has received disparate attention in the past few decades. While the primary focus has been on studying the importance of Fe and Mg during systemic infection, research on the metal homeostasis during enteritis, especially for the biological metals like Zn, Cu, Mn, Mo, Co and Ni, has been neglected. Additionally, contradicting conclusions have left some open questions. As discussed in the sections above, various mouse lines, diverse *Salmonella* genotypes and differing experimental designs across decades of work have led to less-than-congruous outcomes. While most infection studies utilise one of two rather similar and commonly studied *Salmonella* strains (ATCC14028 and SL1344) (Branchu *et al*. 2018), some differences do exist which can affect observed phenotypes (Lopez *et al*. 2012). Strains ATCC14028 and SL1344 were originally isolated from avian and bovine hosts, respectively, though do demonstrate broad host range virulence (Branchu *et al*. 2018). However, other *Salmonella* strains (e.g. clade ST313) are thought to be more human host adapted and are epidemic in sub-Saharan Africa (Kingsley *et al*. 2009; Okoro *et al*. 2012; Branchu *et al*. 2018). Critically, significant differences in pathogenicity between ST313 isolates and SL1344 (and other closely related strains) have been demonstrated (Parsons *et al*. 2013; Carden *et al*. 2017; Ramachandran *et al*. 2017). Therefore, it is important to validate major results in various *Salmonella* genotypes, especially those identified as emerging epidemic threats, in order to broaden our understanding of metal homeostasis and *Salmonella* physiology and translate this knowledge into clinical application. Furthermore, with broadening access to advanced genetic engineering systems (Lanigan *et al*. 2020), a unified understanding of *Salmonella* metal homeostasis is on the horizon. Indeed, comparing mouse lines with unique mutations to study host immune mechanisms' impact on *Salmonella* physiology, instead of comparing mouse lines with various genetic differences, will provide a more complete and unified understanding of relevant host immune function. Finally, focusing on the heterogeneous complexity within host tissues (Lenaerts *et al*. 2015; Kreibich and Hardt 2015; Bumann and Cunrath 2017) will help the field to converge on a comprehensive understanding of *Salmonella* metal homeostasis and its crucial role in pathogenesis.
